# Supplementary material for: A Water-Stable Zn-MOF Used as Multiresponsive Luminescent Probe for Sensing Fe3+/Cu2+, Trinitrophenol and Colchicine in Aqueous Medium
Source: Materials (Basel). 2022 Oct 9;15(19):7006. doi: 10.3390/ma15197006 (PMC9570702; doi:10.3390/ma15197006)
Supplement: Supplementary file 1 [file materials-15-07006-s001.zip › materials-1929115-supplementary.pdf]

materials

MDPI

Electronic Supplementary Information (ESI) for:

A Water-Stable Zn-MOF Used as Multiresponsive Luminescent Probe for Sensing Fe^{3+}/Cu^{2+}, Trinitrophenol and Colchicine in Aqueous Medium

Xiaojing Zhou, Lili Liu, Yue Niu, Mingjun Song, Yimin Feng and Jitao Lu * and Xishi Tai *

School of Chemical & Chemical Engineering and Enviromental Engineering ,Weifang University, Weifang 261061, China

Single crystals structure determination

The data collection and structural analysis were performed on a Rigaku RAXIS-RAPID equipped with a narrow-focus, 5.4 kW sealed tube X-ray source (graphite-monochromated Mo Kα radiation, λ = 0.71073 Å). The data were collected at a temperature of 20 ± 2 °C. The data processing was accomplished with the PROCESS-AUTO processing program. The structures were solved with the direct methods of SHELXL crystallographic software package and refined on F^2 by full-matrx least square techniques. All non-hydrogen atoms of the compound was refined with anisotropic thermal parameters. All hydrogen atoms of the organic molecule were geometrical placed and added to the structure factor calculation. CCDC-1876154 contain the supplementary crystallographic data for this paper. The atomic coordinates for this structure has been deposited with the Cambridge Crystallographic Data Centre. The coordinates can be obtained from the Cambridge Crystallographic Data Centre (CCDC)-https://www.ccdc.cam.ac.uk/.

Table S1. Selected bond distances (Å) and angles (°) for the compound.

**Zn1-O6**	**2.059 (2)**	**Zn5-O6**	**2.130 (2)**
Zn1-O7	2.116 (2)	Zn5-O6i	2.011 (2)
Zn1-O4	2.065 (2)	Zn5-O3i	2.102 (2)
Zn1-O2i	2.137 (2)	Zn5-O1	1.987 (2)
Zn1-O8	2.151 (4)	Zn5-O5B	1.928 (13)
Zn1-N2	2.170 (3)	Zn5-O5A	2.020 (7)
O6-Zn1-O7	96.66 (9)	O4-Zn1-N2	85.25 (10)
O6-Zn1-O4	93.77 (9)	O2i-Zn1-O8	171.69 (14)
O6-Zn1-O2i	91.49 (9)	O2i-Zn1-N2	84.19 (10)
O6-Zn1-O8	92.58 (13)	O8-Zn1-N2	91.93 (13)
O6-Zn1-N2	175.35 (10)	O6i-Zn5-Zn5i	43.42 (6)
O7-Zn1-O2i	85.46 (10)	O6-Zn5-Zn5i	40.47 (6)
O7-Zn1-O8	86.88 (13)	O6i-Zn5-O6	83.89 (9)
O7-Zn1-N2	84.71 (9)	O6i-Zn5-O3i	95.18 (10)
O4-Zn1-O7	168.63 (9)	O6i-Zn5-O5A	133.6 (3)
O4-Zn1-O2i	98.81 (12)	O3i-Zn5-Zn5i	138.18 (8)
O4-Zn1-O8	88.15 (14)	O3i-Zn5-O6	173.04 (10)

Symmetry codes: (i) −*x*+1, −*y*+1, −*z*+1; (ii) *x*−1/2, −*y*+1/2, *z*−1/2; (iii) −*x*+1, *y*, −*z*+1/2; (iv) −*x*+3/2, *y*−1/2, −*z*+3/2; (v) *x*+1/2, −*y*+1/2, *z*+1/2; (vi) −*x*+3/2, *y*+1/2, −*z*+3/2.

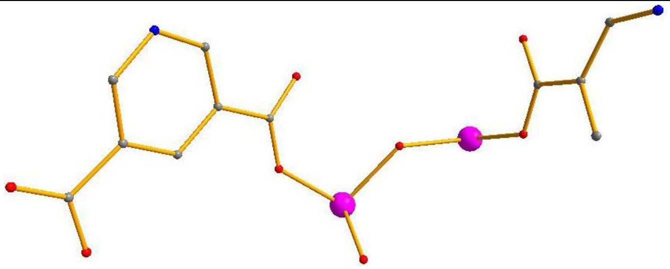

Figure S1. The asymmetric unit of the compound.

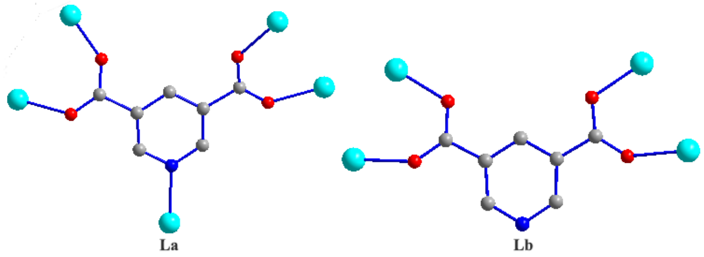

Figure S2. The coordination modes of L_a and L_b.

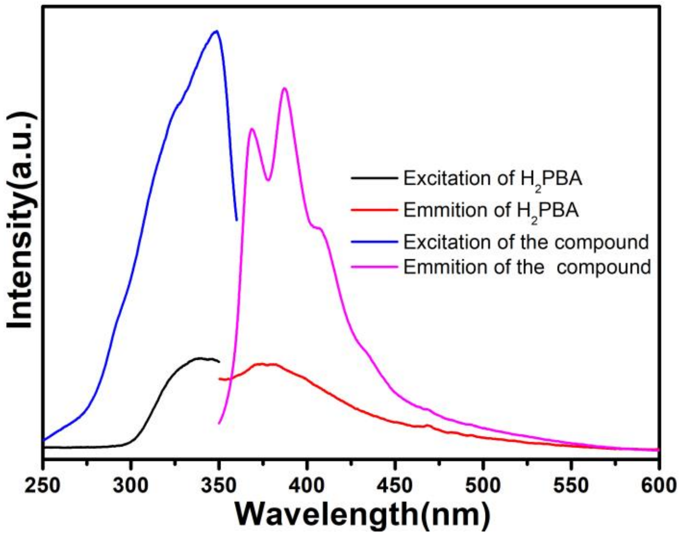

Figure S3. The solid state luminescence of the compound and H_2PBA.

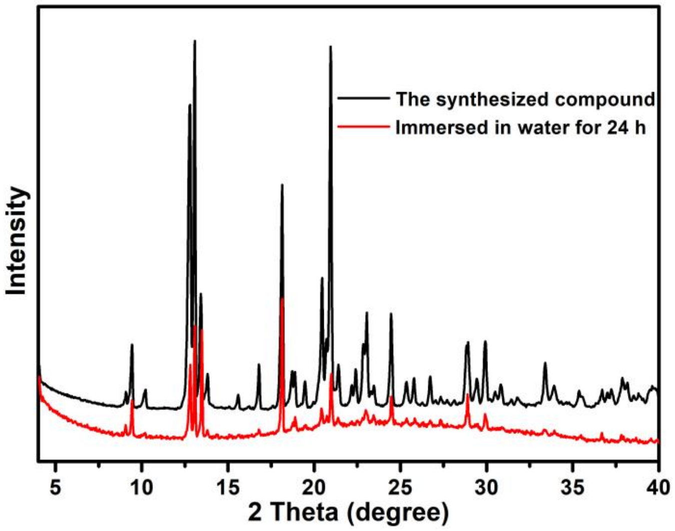

Figure S4. The PXRD of the compound and after immersing in distilled water for 24 h.

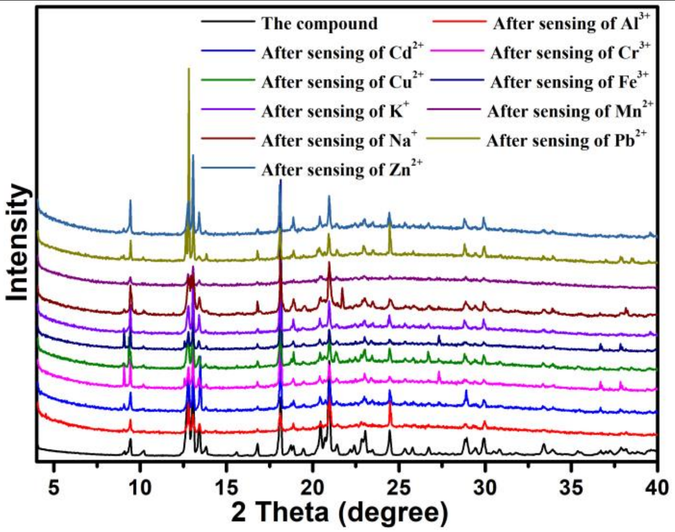

Figure S5. The PXRD of the compound and after sensing of the metal ions.

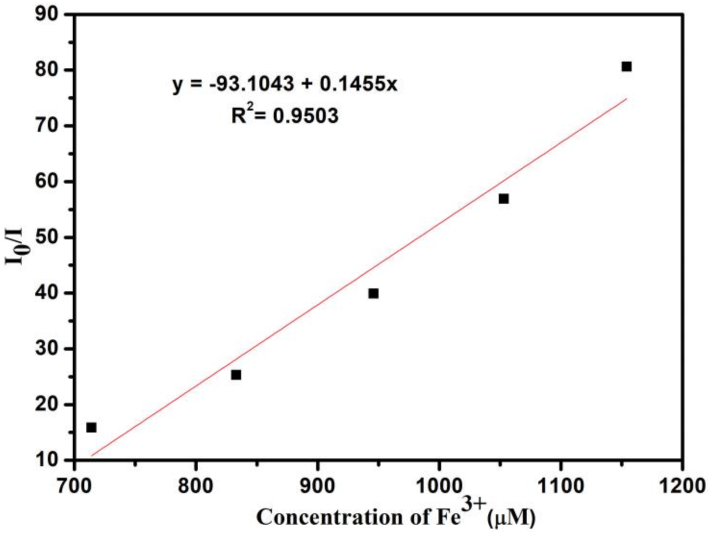

Figure S6. S—V plot for luminescence intensity of the compound upon incremental addition of Fe^{3+} (5× 10^{-3} M) in distilled water.

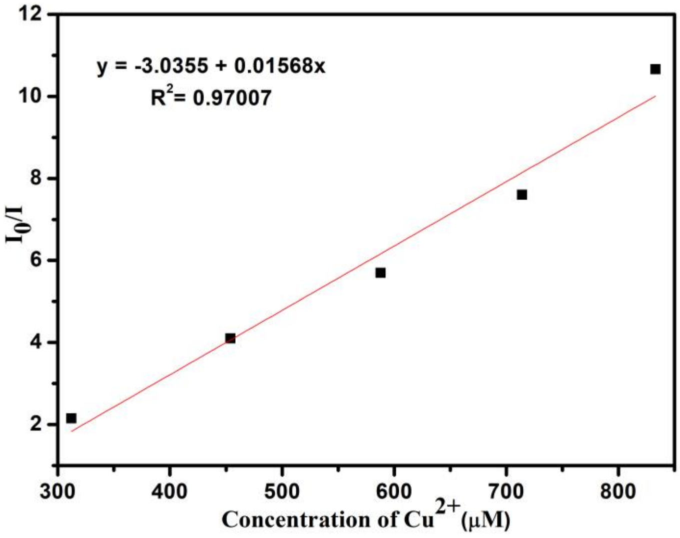

Figure S7. S—V plot for luminescence intensity of the compound upon incremental addition of Cu^{2+} (5× 10^{-3} M) in.

distilled water

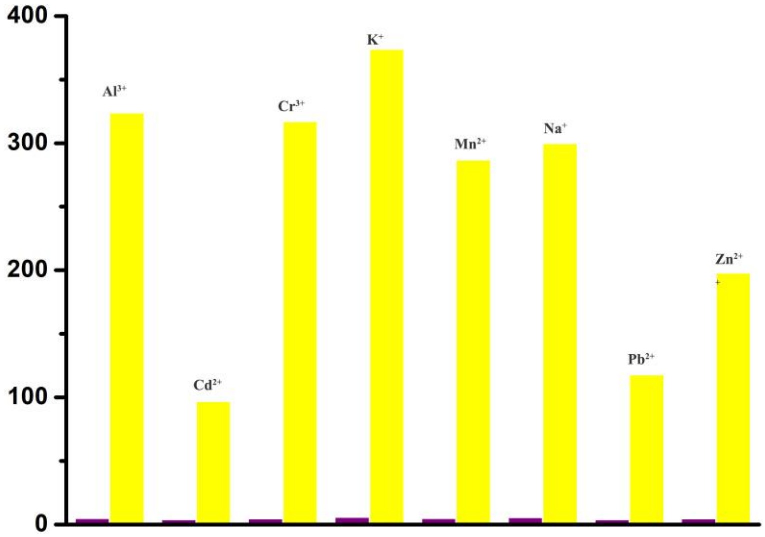

Figure S8. Anti—interference performance of Fe^{3+} (10^{-2} M) in the presence of different metal ions of the compound.

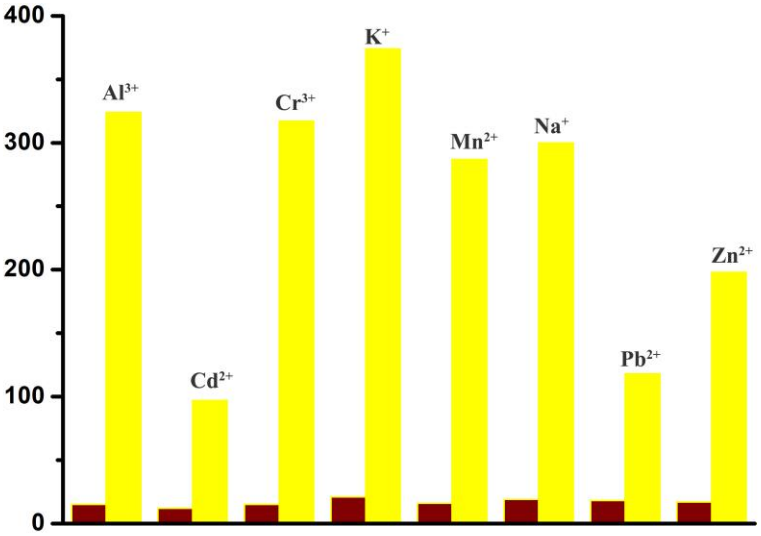

Figure S9. Anti—interference performance of Cu^{2+} (10^{-2} M) in the presence of different metal ions of the compound.

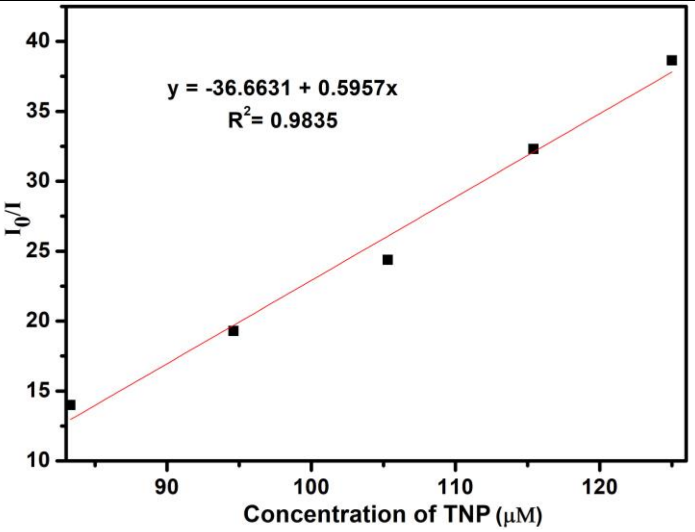

Figure S10. S—V plot for luminescence intensity of the compound upon incremental addition of ~~TNP~~ trinitrophenol (5× 10^{-4} M) in distilled water.

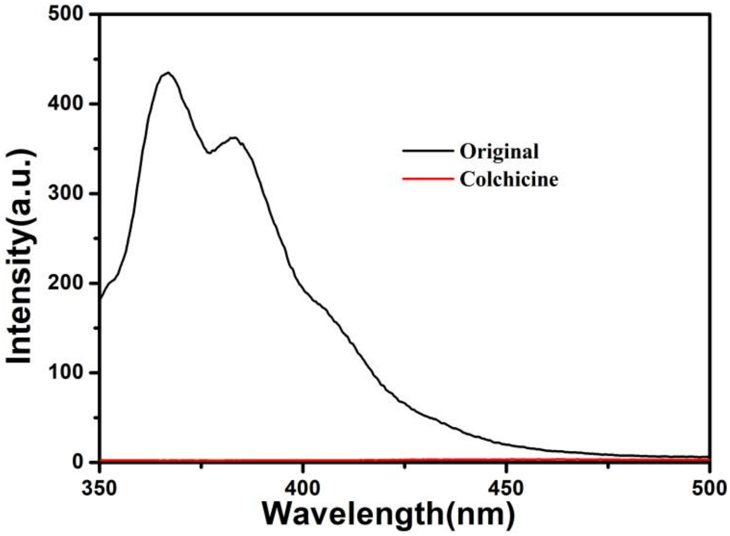

Figure S11. Fluorescence spectral of the compound in water (Black); Fluorescent spectral of the compound towards colchicine (1 × 10^{-2} M) (Red).

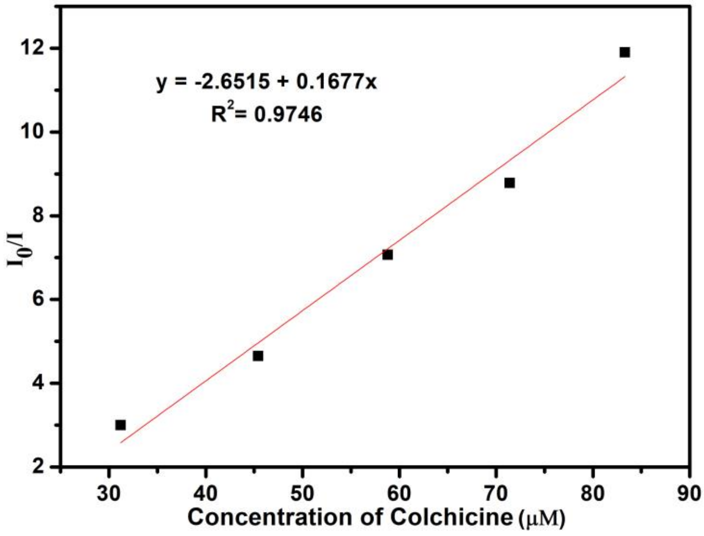

Figure S12. S—V plot for luminescence intensity of the compound upon incremental addition of colchicine.

(5×10^{-4} M) in distilled water

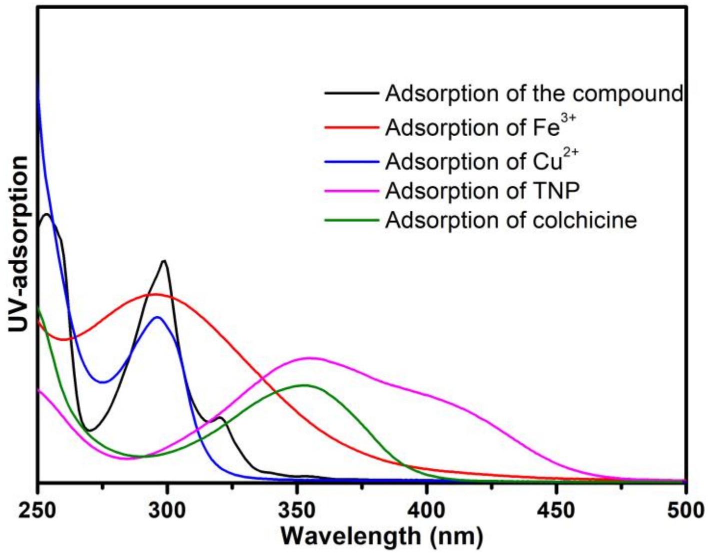

Figure S13. The UV absorption of Fe^{3+}, Cu^{2+}, TNP and colchicine.

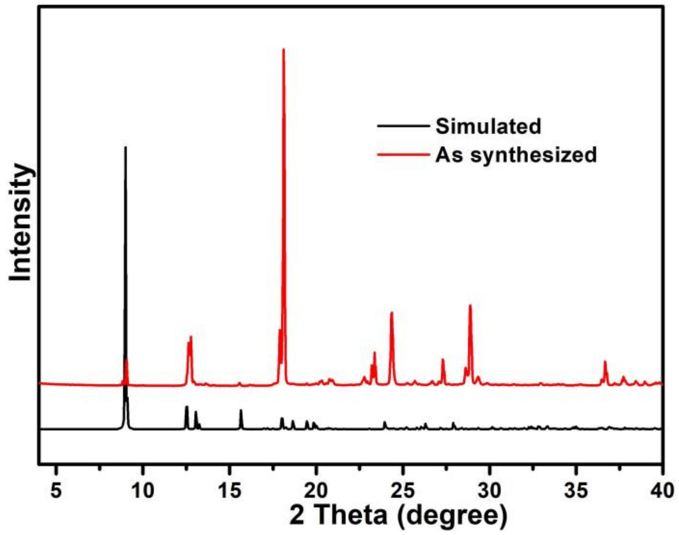

Figure S14. The PXRD of the compound.

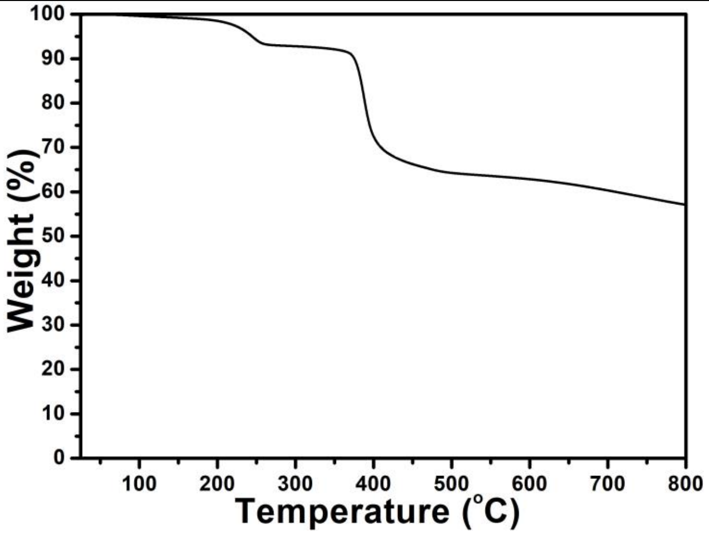

Figure S15. The TGA of the compound.

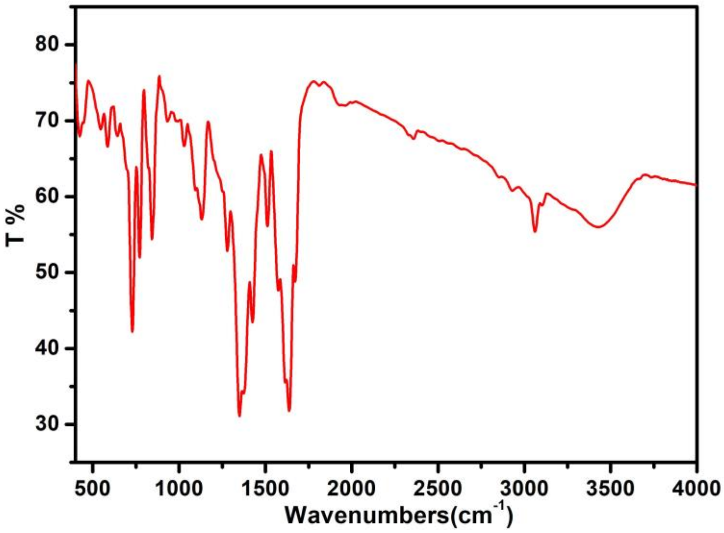

Figure S16. IR of the compound.
